# The prognostic biological markers of immunotherapy for non-small cell lung cancer: current landscape and future perspective

**DOI:** 10.3389/fimmu.2023.1249980

**Published:** 2023-09-11

**Authors:** Shuai Liang, Hanyu Wang, Haixia Tian, Zhicheng Xu, Min Wu, Dong Hua, Chengming Li

**Affiliations:** ^1^ Department of Oncology, The Affiliated Wuxi People’s Hospital of Nanjing Medical University, Wuxi, China; ^2^ Wuxi School of Medicine, Jiangnan University, Wuxi, China; ^3^ Suzhou Cancer Center Core Laboratory, The Affiliated Suzhou Hospital of Nanjing Medical University, Suzhou, China; ^4^ Department of Radiation Oncology, Shandong Cancer Hospital and Institute, Shandong First Medical University and Shandong Academy of Medical Sciences, Jinan, China

**Keywords:** non-small cell lung cancer (NSCLC), immunotherapy, prognostic biological markers, anti-PD-(L)1, review

## Abstract

The emergence of immunotherapy, particularly programmed cell death 1 (PD-1) and programmed cell death ligand-1 (PD-L1) produced profound transformations for treating non-small cell lung cancer (NSCLC). Nevertheless, not all NSCLC patients can benefit from immunotherapy in clinical practice. In addition to limited response rates, exorbitant treatment costs, and the substantial threats involved with immune-related adverse events, the intricate interplay between long-term survival outcomes and early disease progression, including early immune hyperprogression, remains unclear. Consequently, there is an urgent imperative to identify robust predictive and prognostic biological markers, which not only possess the potential to accurately forecast the therapeutic efficacy of immunotherapy in NSCLC but also facilitate the identification of patient subgroups amenable to personalized treatment approaches. Furthermore, this advancement in patient stratification based on certain biological markers can also provide invaluable support for the management of immunotherapy in NSCLC patients. Hence, in this review, we comprehensively examine the current landscape of individual biological markers, including PD-L1 expression, tumor mutational burden, hematological biological markers, and gene mutations, while also exploring the potential of combined biological markers encompassing radiological and radiomic markers, as well as prediction models that have the potential to better predict responders to immunotherapy in NSCLC with an emphasis on some directions that warrant further investigation which can also deepen the understanding of clinicians and provide a reference for clinical practice.

## 1 Introduction

Lung carcinoma is widely acknowledged as being the foremost reason for mortality connected to neoplasms in both the U.S. and China, which is with around 85% of pulmonary neoplasms classified as non-small cell lung carcinomas (NSCLCs) (1, 2). And the advent of immunotherapeutic agents targeting programmed cell death 1 (PD-1) and programmed cell death ligand-1 (PD-L1), also known as one of immunoregulatory checkpoint inhibitors (ICIs), altered thoroughly the management of patients with progressive or metastatic NSCLC. Furthermore, the utilization of PD-1 inhibitors (Nivolumab, Pembrolizumab) and PD-L1 inhibitors (Atezolizumab) has been endorsed by the U.S. Food and Drug Administration (FDA) in terms of patients’ treatments with advanced NSCLC experiencing disease progression in or post initial-line therapy due to their superior disease progression-free survival (PFS) and overall survival (OS) outcomes in comparison to the conventional chemotherapy comparator (3–6). Nevertheless, the favorable response to PD-1/PD-L1 inhibitors is not universal, as merely 20 - 40% of patients exhibit a response, fewer achieve long-term disease remission, and even some patients could experience immune hyperprogression at the early stage of immunotherapy (7–9). Given the exorbitant costs and potential for severe adverse effects associated with ICIs, it becomes imperative to find patients with the most likely to derive therapeutic advantage from such treatments and enhance the efficacy of ICIs for precise therapeutic interventions. Consequently, the pursuit of reliable and effective biological markers for assessing the reaction to anti-PD-1/PD-L1 immunotherapy has become the focal point of oncoimmunology investigations in NSCLC patients. Although biological markers including PD-L1 expression levels, tumor mutational burden (TMB), hematological biological markers, and composite biological markers are presently under scrutiny as potential indicators for assessing the reaction to ICIs in NSCLC patients, none have gained widespread and accurate clinical utilization. Thus, in this review, we comprehensively examine the current landscape of biological markers for anti-PD-1/PD-L1 immunotherapy in NSCLC with an emphasis on some directions that warrant further investigation which can also deepen the understanding of clinicians and provide a reference for clinical practice.

## 2 Single biological marker

### 2.1 PD-L1 expression

Currently, PD-L1 expression serves as one of the highest extensively utilized biological markers to predict the ICIs efficiency in NSCLC. As per the guidelines by the National Comprehensive Cancer Network (NCCN), anti-PD-L1 immunotherapy (Atezolizumab) is suggested as first-line treatment for NSCLC patients exhibiting PD-L1 expression ≥ 50% or as second-line treatment for those with PD-L1 expression ≥ 1% (3). PD-1, an immunoregulatory checkpoint receptor expressed on activated T cells, including tumor-infiltrating lymphocytes, plays a pivotal role in immune evasion when it interacts with its ligands, PD-L1 and PD-L2, resulting in the suppression of cytotoxic T cell responses (10–13). Upregulation of PD-L1 is frequently observed on tumor cell surfaces, with PD-L1 expression reported in approximately 20% to 40% of NSCLC cases (14). Extensive investigations have delved into the connection between PD-L1 expression levels and immunotherapy efficacy, although the predictive value of PD-L1 remains controversial (**Table 1**) (15–20, 22–30, 34, 35). The KEYNOTE-024 trial showed that Pembrolizumab exhibited better OS in comparison to chemotherapy in patients displaying high PD-L1 expression (≥ 50%) (21). For PACIFIC trials, unresectable stage III NSCLC patients who completed radiotherapy and chemotherapy were administered Durvalumab for up to one year (36). It was observed that Durvalumab improved survival in patients with PD-L1 expression ≥ 25%. However, patients with low PD-L1 expression (< 1%) did not derive significant benefits from immune consolidation therapy. A meta-analysis involving 12 randomized clinical trials encompassing 6,932 patients with locally advanced NSCLC who received PD-1/PD-L1 inhibitors indicated that individuals with high PD-L1 expression levels exhibited longer PFS, OS, and overall response rate (ORR) in comparison to those with low PD-L1 levels (37). Of note, patients with PD-L1 expression ≥ 1% demonstrated a significant improvement in ORR, whilst little statistically significant disparity had been noted among patients with PD-L1 expression < 1% (*P* = 0.12).

**Table 1:** Clinical outcomes of NSCLC patients treated with anti-PD-1/anti-PD-L1 agents according to the expression of PD-L1.

Study	Patients (N)	Drug	Assay	Cut-off value	(m)PFS (months)	(m)OS (months)	ORR (%)		Ref.
POPLAR	287	Atezolizumab **vs.** Docetaxel	SP142	50% (TC) or 10% (IC)	7.8 vs. 3.9	19.9 vs. 11.1	38 vs. 13		(3, 15)
KEYNOTE 010	442	Pembrolizumab (2 or 4 mg/kg) **vs.** Docetaxel	22C3	50% (TPS)	5.0 or 5.2 vs. 4.1	14.9 or 17.3 vs. 8.2	30 or 29 vs. 8		(4)
Based on KEYNOTE 010	1033	Pembrolizumab **vs.** Docetaxel	22C3	50% (TPS) 1% (TPS) 1-49% (TPS)	5.3 vs. 4.1 3.9 vs. 4.1 2.6 vs. 4.1	17.1 vs. 8.2 11.9 vs. 8.6 10.2 vs. 8.7	32.4 vs. 8.6 20.9 vs. 9.3 NA		(4, 16)
Checkmate 017	272	Nivolumab **vs.** Docetaxel	28-8	NA	3.5 vs. 2.8	9.2 vs. 6.0	20 vs. 9		(5)
OAK	850	Atezolizumab **vs.** Docetaxel	SP142	1% (TC or IC) 50% (TC or IC)	Similar	12.6 vs. 8.9 20.5 vs. 8.9	8 vs. 11 31 vs.11		(6)
Based on Checkmate 017	272	Nivolumab **vs.** Docetaxel	28-8	1% (TPS) 50% (TPS)	NA	NA	17 vs. 11 29 vs. 10		(5, 17)
Based on OAK	850	Atezolizumab **vs.** Docetaxel	SP142	1% (TC or IC) 50% (TC or IC)	NA	20.5 vs. 9.7 11.8 vs. 8.9	NA NA		(6, 15)
Checkmate 057	457	Nivolumab **vs.** Docetaxel	28-8	1% (TPS) 5% (TPS) 10% (TPS)	4.2 vs. 4.5 5.0 vs. 3.8 5.0 vs. 3.7	17.7 vs. 9.0 19.4 vs. 8.1 19.0 vs. 8.0	38 vs. 15 34 vs. 11 32 vs. 10		(18)
Based on Checkmate 057	582	Nivolumab **vs.** Docetaxel	28-8	1% (TPS) 50% (TPS)	NA	NA	31 vs. 12 41 vs. 7		(17, 18)
CheckMate 078	252	Nivolumab **vs.** Docetaxel	28-8	≥1% (TPS) <1% (TPS)	NA	12.3 vs. 7.9 11.4 vs. 10.2	NA		(19)
	205								
KEYNOTE 042	1274	Pembrolizumab **vs.** Chemotherapy	22C3	1% (TPS) 20% (TPS) 50% (TPS)	5.4 vs. 6.5 6.2 vs 6.6 7.1 vs. 6.4	16.7 vs. 12.1 NA NA	27 vs. 27 33 vs. 29 39 vs. 32		(20)
KEYNOTE 024	305	Pembrolizumab **vs.** Chemotherapy	22C3	50% (TC)	10.3 vs. 6.7	NA	44.8 vs. 27.8		(21)
Based on KEYNOTE 010 KEYNOTE 024 KEYNOTE 042	264 (elderly)	Pembrolizumab **vs.** Chemotherapy	22C3	1% (TPS) 50% (TPS)	NA	15.7 vs. 11.7 23.1 vs. 8.3	NA		(4, 20–22)
	2348 (younger)			1% (TPS) 50% (TPS)		14.6 vs. 11.1 19.2 vs.11.9			
MYSTIC	488	Durvalumab **vs.** Durvalumab **+** Tremelimumab **vs.** Chemotherapy	SP263	25% (TC)	NA	11.1 vs 10.5 vs. 13.3 (25-49%) 18.3 vs. 15.2 vs. 12.7 (50%)	35.6 vs. 34.4 vs. 37.7		(23)
CheckMate 568	252	Nivolumab + Ipilimumab	28-8	1% (TC)	6.8 vs. 2.8	NA	41 vs. 15		(24)
Checkmate 012	44	Nivolumab **+** Ipilimumab (12 weeks **vs.** 6 weeks)	28-8	1% (TC)	8.1 vs. 10.6	NA	57 vs 57		(25)
EMPOWER-Lung 1	563	Cemiplimab **vs**. Chemotherapy	22C3	50% (TC)	8.2 vs. 5.7	NR vs. 14.2	36.5 vs. 20.6		(26)
Impower 110	554	Atezolizumab **vs.** Chemotherapy	NA	50% (TC) or 10% (IC) 5% (TC or IC) 1% (TC or IC)	NA NA NA	20.2 vs. 13.1 18.2 vs. 14.9 17.5 vs. 14.1	NA NA NA		(27)
CameL	255	Camrelizumab + Chemotherapy **vs.** Chemotherapy	22C3	1% (TPC)	15.4 vs. 9.9	NA	NA		(28)
CheckMate 227	1189 550	Nivolumab + Ipilimumab **vs.** Nivolumab **vs.** Chemotherapy	28-8	≥ 1% (TC) < 1% (TC)	5.1 **vs.** 4.2 **vs.** 5.6 5.1 **vs.** 5.6 **vs.** 4.7	17.1 **vs.** 15.7 **vs.** 14.9 17.4 **vs.** 15.2 **vs.** 12.2	36 **vs.** 28 **vs.** 30 27 **vs.** 38 **vs.** 23		(29, 30)
KEYNOTE 407	207	Pembrolizumab combination **vs.** Placebo combination	22C3	1% (TPS)	7.2 vs. 5.2	14.0 vs. 11.6	49.5 vs. 41.3		(31)
	146			50% (TPS)	8.0 vs. 4.2	NR vs. NR	60.3 vs. 32.9		
GP28328	76	Atezolizumab + Chemotherapy	SP142	1% (TC or IC)	6.0 vs. 5.6	15.0 vs. 12.9	45.2 vs.41.2		(32)
CheckMate 026	423 214	Nivolumab **vs.** Chemotherapy	28-8	5% (TPS) 50% (TPS)	4.2 vs. 5.9 NA	14.4 vs. 13.2 NA	26 vs. 33 34 vs. 39		(33)

NA, not available; NR, not reached.

Even though individuals exhibiting elevated PD-L1 expression exhibited a higher likelihood of responding ICIs, around 10% of PD-L1-negative patients also displayed a reaction to ICIs. Conversely, there were instances where patients with substantial PD-L1 expression exhibited an unresponsive state (4, 5, 31, 32). Within the framework of the KEYNOTE-024 trial, the ORR for PD-L1 ≥ 50% NSCLC patients receiving Pembrolizumab was a mere 44.8%, thereby indicating a considerable proportion of individuals with heightened PD-L1 expression did not manifest a response to this treatment (28). Moreover, the CheckMate-026 trial revealed that a PD-L1 expression level of ≥ 50% failed to serve as a reliable predictor of the effectiveness of Nivolumab in first-line NSCLC treatment (ORR, 34% vs. 39%) (33). And CheckMate 227 also found that Nivolumab plus Ipilimumab increased 5-year survivorship verse chemotherapy regardless of PD-L1 expression (29). One of the most obstacles impeding the clinical implementation of PD-L1 as a biological marker for predicting response to anti-PD-1/PD-L1 could be the absence of standardization in PD-L1 testing. Presently, the FDA has sanctioned the utilization of three PD-L1 immunohistochemistry (IHC) assays as companion diagnostics: Dako 22C3 (22C3) for Pembrolizumab in patients with diverse solid tumors, including NSCLC; Ventana SP142 (SP142) for Atezolizumab in NSCLC patients; and Dako 28-8 (28-8) for the combination of Ipilimumab and Nivolumab in NSCLC patients (38). Then, discrepancies in the findings of various studies could potentially arise from disparities in antibodies, detection methodologies, environmental conditions at the time of analysis, and the threshold values employed to determine PD-L1 expression (39, 40). For instance, the KEYNOTE-028 phase Ib study necessitated PD-L1 expression exceeding 1% on tumor or stromal cells, per measurement by the 22C3 assay, for advanced-stage solid tumors patients who received Pembrolizumab (41). Furthermore, variations in previous treatments administered to patients across different studies could have also contributed to inconsistencies in the research outcomes (42, 43). Last, the expression of PD-L1 is dynamic, influenced by treatments such as chemotherapy, radiotherapy, or chemoradiotherapy, which can influence PD-L1 expression within the tumor (44–46). Consequently, studies encompassing larger cohorts are imperative to validate the significance of PD-L1 as a predictive biological marker for ICI response in NSCLC patients.

### 2.2 TMB

Apart from PD-L1 expression, TMB stands as another extensively studied biological marker for predicting the efficacy of immunotherapy in NSCLC (10, 47). Somatic mutations can induce substantial modifications in protein sequences, resulting in the generation of abnormal proteins. These anomalous proteins can act as novel antigenic peptides, exhibiting a strong affinity for major histocompatibility complexes (MHC) I or MHC II. Thereafter, these neoepitopes are presented on the cell surface, where they are identified as non-self by the immune system, thereby instigating the activation of T lymphocytes that are specific to these neoantigens. Consequently, the immunogenicity of a tumor is intimately associated with the accumulation of somatic mutations within tumor cells (48). Elevated tumor mutational burden (referred to as TMB-H) leads to an augmented production and release of neoantigens, thereby enhancing the recognition and elimination of tumor cells by activated cytotoxic T cells after treatment with ICIs (48). Notably, NSCLC demonstrates a comparatively higher TMB-H than other solid tumor types (49). Consequently, the potential value of TMB as a biological marker for predicting the efficacy of immunotherapy has been extensively investigated in NSCLC patients (**Table 2**) (50–52).

**Table 2:** Clinical outcomes of NSCLC patients treated with anti-PD-1/anti-PD-L1 agents according to the expression of TMB.

Study	Patients (N)	Therapy	TMB cut-off (mut/Mb)	(m)PFS (months)	(m)OS (months)	ORR (%)	Ref.
MYSTIC	809 460	Durvalumab **vs.** Durvalumab **+** Tremelimumab **vs.** Chemotherapy	20 (bTMB) 10 (tTMB)	2.7 vs.4.2 vs. 4.4 3.1 vs. 3.1 vs. 5.1	12.6 vs. 21.9 vs. 10.0 18.6 vs. 16.6 vs. 11.9	NA	(23)
CheckMate 568	98	Nivolumab **+** Ipilimumab	10 (tTMB)	7.1 vs.2.6	NA	44 vs. 12	(24)
CheckMate 026	312	Nivolumab **vs.** Chemotherapy	243 (tTMB and bTMB)	9.7 vs. 5.8	Similar	47 vs. 28	(33)
CheckMate 227	299	Nivolumab **+** Ipilimumab **vs.** Chemotherapy	10 (tTMB and bTMB)	7.2 vs. 5.5	NA	45.3 vs. 26.9	(29, 35)
Based on POPLAR	273	Atezolizumab **vs.** Docetaxel	16 (bTMB)	4.2 vs. 2.9	13.0 vs. 7.4	NA	(3, 50)
Based on OAK	797	Atezolizumab **vs.** Docetaxel	16 (bTMB)	NA	13.5 vs. 6.8	NA	(6, 50)
B-F1RST	119	Atezolizumab	16 (bTMB)	4.6 vs. 3.7	NR vs. 13.1	28.6 vs. 4.4	(51)
Based on CheckMate 012	75	Nivolumab **+** Ipilimumab	158	NR	NA	51 vs. 13	(25, 52)

NA, not available; NR, not reached.

During the phase III clinical trial also known as CheckMate-026, researchers conducted a retrospective assessment of the TMB in 312 patients diagnosed with NSCLC who received either Nivolumab or chemotherapy through whole-exome sequencing (33). Among the patients with high TMB (≥ 243 mut/Mb), Nivolumab exhibited a superior ORR of 47% in comparison to 28% in the chemotherapy group, along with a longer PFS of 9.7 vs. 5.8 months. *Gandara* et al. conducted a retrospective analysis of blood-based tumor mutational burden (bTMB) in patients participating in the OAK and POPLAR studies. They confirmed that a bTMB of ≥ 16 mut/Mb could serve as a predictive factor for OS and PFS in NSCLC patients treated with second-line Atezolizumab (3, 6, 50). Subsequently, a prospective study called B-F1RST categorized NSCLC patients receiving Atezolizumab into two groups based on their bTMB levels: a high bTMB group (bTMB-H) with ≥ 16 mut/Mb, and a low bTMB group (bTMB-L) with < 16 mut/Mb (51). The bTMB-H group exhibited a significantly higher ORR of 28.6% in comparison to 4.4% in the bTMB-L group (*P* = 0.0002). Additionally, patients in the bTMB-H group experienced a longer PFS of 4.6 months in comparison to 3.7 months in the bTMB-L group (*P* = 0.12). Similarly, a positive correlation was observed between bTMB-H and improved OS (*P* = 0.48), despite that the correlations between OS and PFS yielded little statistical significance. Furthermore, the MYSTIC study, a phase III clinical trial involving NSCLC patients, demonstrated that Durvalumab, when in comparison to chemotherapy, did not provide a substantial overall survival benefit in patients with high bTMB (23). Another trial, CheckMate-227, compared the effectiveness of Nivolumab plus Ipilimumab to that of dual-drug chemotherapy containing platinum in patients with TMB ≥ 10 mut/Mb (29). In the TMB-H group, patients treated with the combination of Ipilimumab and Nivolumab showed higher ORR (45.3% vs. 26.9%) and a lower hazard ratio for PFS (0.58, *P* < 0.001) in comparison to those treated with chemotherapy. Descriptive analyses of overall survival in the CheckMate-227 trial revealed an improvement in patients with high TMB and low TMB (< 10 mut/Mb) who were treated with Nivolumab plus Ipilimumab. A meta-analysis encompassing 14,395 NSCLC patients demonstrated that the combination of programmed cell death ligand 1 (PD-L1) expression levels and TMB served as a reliable biological marker for predicting response to immunotherapy. The area under the curve (AUC) for 1-year and 3-year PFS exceeded 0.8 (53). Notably, the findings from CheckMate-026 indicated a lack of significant association between TMB and PD-L1 expression level (r = 0.059) (33).

However, it is important to note that TMB testing is a costly endeavor. At present, TMB determination primarily relies on two methods: tissue-based next-generation sequencing (FoundationOne) and whole exome sequencing (WES). WES necessitates samples with high DNA quality and a substantial tumor cell fraction, making high-quality samples a prerequisite for TMB testing. In the CheckMate-227 study, only 57.7% of the samples were available for TMB analysis, and a mere 10.3% of those patients tested positive for TMB (30, 35). Additionally, the variability in gene sequences and cutoff values employed by different studies to detect TMB using various panels contributes to inconsistencies in the results (54, 55). For instance, in the CheckMate-568 study, the ability of TMB = 10 mut/Mb to predict the effectiveness of Nivolumab plus Ipilimumab in NSCLC patients was ascertained via receiver operating characteristic (ROC) curve assessment (24). It is worth highlighting that the sample used for TMB analysis is obtained from a specific part of the tumor. Consequently, the molecular detection method based on biopsy is inevitably influenced by sampling deviation owing to intratumoral heterogeneity (56). In light of these factors, the combination of PD-L1 expression and TMB appears to hold more promise in clinical practice. Nonetheless, further prospective, randomized, controlled trials are warranted to ascertain the reliability of TMB as a biological marker for predicting the response to ICIs.

### 2.3 Hematological biological markers

#### 2.3.1 CD8^+^ T cells

The key to eliciting the host’s immune response against malignant cells lies in the recognition of novel antigens by T cell receptors (TCR) (57). Consequently, biological markers founded on TCR may hold the potential to forecast the response to immunotherapy (**Table 3**). The sequencing of TCR lineages can be readily performed utilizing peripheral blood samples, thereby affording a non-intrusive approach for prognosticating the response to ICIs. *Han* and colleagues performed a sequencing analysis of PD-1^+^CD8^+^ TCRs in the peripheral blood of 25 patients diagnosed with NSCLC undergoing ICI treatment. The study revealed that patients exhibiting heightened diversity of PD-1^+^CD8^+^ TCRs before immunotherapy demonstrated superior treatment response and PFS in comparison to those with diminished TCR diversity (6.4 vs. 2.5 months, *P* = 0.021) (58). These findings were subsequently validated in a cohort comprising 15 patients. Importantly, the sensitivity and specificity of pre-immunotherapy PD-1^+^CD8^+^ TCR diversity in predicting PFS were determined to be 0.87 and 0.94, respectively. Patients with elevated clonality of PD-1^+^CD8^+^ TCRs following immunotherapy displayed prolonged PFS in contrast to those with diminished clonality (7.3 vs. 2.6 months, *P* = 0.002). Similarly, the proliferative response of peripheral blood PD-1^+^CD8^+^ T cells (quantified as the fold change in the percentage of Ki-67+ cells seven days post-treatment) proved to be a valuable surrogate biological marker for predicting the response to ICIs in NSCLC patients (59). Additionally, *Kamphorst et al.* ascertained that within the cohort of patients with advanced NSCLC, PD-1^+^CD8^+^ T cell responses were either delayed or absent in 70% of those experiencing disease progression, while 80% of the patients who positively responded to immunotherapy exhibited PD-1^+^CD8^+^ T cell responses within four weeks of treatment initiation (78). Given the paucity of clinical evidence, the amalgamation of TCR diversity and clonality in PD-1^+^CD8^+^ T cells derived from peripheral blood might serve as a non-invasive biological marker in conjunction with PD-L1 levels and TMB for predicting the response to immunotherapy and prognosticating outcomes in NSCLC patients.

**Table 3:** Clinical outcomes of NSCLC patients treated with anti-PD-1/anti-PD-L1 agents according to hematological biological markers.

Characteristics	Patients (N)	Category	(m)PFS (months)	(m)OS (months)	Ref
PD-1^+^CD8^+^ T cells	25	High **vs.** Low	6.4 vs. 2.5	NR vs. 6.9	(58)
	19	Increased **vs.** Decreased	7.3 vs. 2.6	NR vs. 7.5	
Ki-67^+^_D7/D0_	33	≥ 2.8 **vs.** < 2.8	6.0 vs. 1.4	13.8 vs. 2.0	(59)
	46	≥ 2.8 **vs.** < 2.8	10.9 vs. 2.1	NR vs. 7.0	
ctDNA	15	at w8 **vs.** persistently detectable	11 vs. 2	NA	(60)
ctDNA and NLR	45	increased >20% at w6 **vs.** decreased	NA	5.7 vs. 14.2	(61)
		increased >20% at w6 **vs.** decreased	NA	8.7 vs. 14.6	
		combined increased >20% of both ctDNA and NLR at w6 **vs.** others	NA	5.8 vs. 15.5	
ctDNA	28	Responders **vs.** non-responders A ctDNA response was associated with superior PFS and OS			(62)
ANC, ALC, AEC	134	Low **vs.** High Low ANC, high ALC, and high AEC were significantly and independently associated with better PFS and OS			(63)
ANC : ALC ratio, M:L ratio	157	Increased baseline **vs.** decreased	Increased baseline ANC : ALC ratio and M:L ratio before initiation of anti-PD1 antibodies were associated with poor PFS and OS		(64)
NLR PLR	187	Low levels (<5) **vs.** others	7.0 vs. 4.0	15.0 vs. 6.0	(65)
		levels below 200 **vs.** others	7.0 vs. 4.0	15.0 vs. 11.0	
NLR PLR SII	44	≤ 3.07 **vs.** > 3.07	6.7 vs. 3.9	19.8 vs. 8.9	(66)
		≤ 144 **vs.** > 144	28.5 vs. 10.5	6.9 vs. 3.9	
		≤ 603.5 **vs.** > 603.5	6.9 vs. 2.4	19.8 vs. 8.9	
NLR ALI	201	< 4 **vs.** ≥ 4	3.5 vs. 1.5	NA	(67)
		< 18 **vs.** ≥ 18	3.7 vs. 1.4	NA	
NLR	52	Higher **vs.** Lower Elevated pre-treatment NLR was associated with worse OS and response rates			(68)
NLR	175	< 5 **vs.** ≥ 5	2.8 vs. 1.9	8.4 vs. 5.5	(81)
NLR	52	< 5 **vs.** ≥ 5	3.3 vs. 1.7	NR vs. 4.2	(69)
NLR baseline NLR at w8 ANC at w8	88	≤ 4 **vs.** > 4 Baseline NLR ≤ 4 and lower NLR and ANC ≤ 4 **vs.** > 4 during treatment might correlate with disease Lower **vs.** Higher control and treatment response			(70)
NLR at w6	54	< 5 **vs.** ≥ 5	6.1 vs. 1.3	14.0 vs. 2.1	(71)
NLR PLR	119	≤ 5 **vs.** NLR > 5	18.82 vs. 6.86 (mean)	40.59 vs. 19.42 (mean)	(72)
		High **vs.** Low	11.01 vs. 15.96 (mean)	22.05 vs. 38.47 (mean)	
dNLR	466	≤ 3 **vs.** > 3 A dNLR greater than 3 was independently associated with OS			(73)
dNLR	63	≤ 3 **vs.** > 3 High dNLR was the independent statistically significant parameter associated with PFS and OS			(74)
CEA	189	≥13.8 **vs.** < 13.8 Higher CEA was associated with inferior PFS			(75)
CYFRA 21-1	50	≥2.2 **vs.** < 2.2	155 vs. 51.5 (days)	NA	(76)
CEA	84	≥5 **vs.** < 5	1.7 vs. 2.7	5.6 vs. 12.1	(77)
		Reduction≥20% after 4 cycles of nivolumab **vs.** others	7.1 vs. 1.9	15 vs. 9.9	
CYFRA 21-1		≥3.3 vs. < 3,3	NA	5.6 vs. 13.2	
		Reduction≥20% after 4 cycles of nivolumab **vs.** others	7.9 vs. 1.9	14.6 vs. 10	

Ki-67^+^_D7/D0_, Ki-67^+^ cells among PD-1^+^CD8^+^ T cells 7days after the first dose; NLR, neutrophil-to-lymphocyte radio; ANC, absolute neutrophil count; ALC, absolute lymphocyte count; AEC, absolute eosinophil count; ANC : ALC radio, ANC to ALC radio; M:L radio, myeloid to lymphoid radio; SII = platelet count **×** neutrophil count**/**lymphocyte count; ALI, inflammation index (which was calculated as BMI**×**ALB**/**NLR); dNLR, derived neutrophils**/**(leukocytes minus neutrophils) ratio; CEA, carcinoembryonic antigen; W6, week 6; W8, week 8.

#### 2.3.2 Circulating tumor DNA

Circulating tumor DNA (ctDNA) is a potent prognostic biological marker due to its resemblance to DNA obtained from solid tumor biopsies, often referred to as a “liquid tissue biopsy” (79–81). Many studies have demonstrated that alterations in ctDNA levels during chemotherapy are correlated with treatment response across various tumor types, including NSCLC (**Table 3**) (60–62, 82–86). In a study encompassing a cohort of five patients afflicted with metastatic melanoma who underwent treatment with Ipilimumab, alterations in ctDNA levels during therapy exhibited concurrence with the outcomes derived from imaging-based evaluation of treatment response (86). Hence, modifications in ctDNA levels during immunotherapy hold the potential to serve as a biological marker for monitoring immunotherapy response and prognosticating patient outcomes. Moreover, there exists evidence supporting the utility of ctDNA levels as a biological marker in foretelling the response to immunotherapy among NSCLC patients. *Cabel et al.* executed a study involving 15 patients diagnosed with advanced cancer, ten of whom had NSCLC (60). Pre-treatment blood specimens were procured and subsequently, eight weeks following the initiation of immunotherapy, ctDNA levels were evaluated. The findings demonstrated a positive correlation between tumor regression 8 weeks post-treatment and a decline in ctDNA levels (r = 0.86, *P* = 0.002). Furthermore, ctDNA levels assessed eight weeks after treatment exhibited a significant association with both PFS (*P* < 0.001) and OS (*P* = 0.004). In another study involving 45 NSCLC patients who underwent Nivolumab immunotherapy, *Passiglia et al.* demonstrated that a 20% increase in ctDNA levels observed 6 weeks after treatment was indicative of inferior OS (median OS, 5.7 vs. 14.2 months, *P* < 0.001) and time to progression (TTP) (3.3 vs. 10.2 months, *P* < 0.001) (61). Subsequent investigations further validated the capacity of ctDNA level alterations in predicting response to immunotherapy, emphasizing that changes in ctDNA levels could predict response earlier than assessments based on imaging (24.5 vs. 72.5 days) (62). Nevertheless, it is noteworthy that the sample sizes of the aforementioned studies were relatively small, and there exists a lack of uniformity in the threshold for ctDNA change and the timing of evaluating molecular response across each study. Consequently, it is imperative to establish a standardized framework for monitoring dynamic changes in ctDNA. The potential to proactively modify patients’ survival outcomes based on dynamic changes in ctDNA before observable imaging progression, as well as the prospect of employing liquid biopsy as a replacement for traditional imaging in the evaluation of early treatment response to immunotherapy, necessitate further deliberation.

#### 2.3.3 Blood cell count

The changes of lymphocytes and neutrophils in peripheral blood can reflect the immune state of the body and can predict the curative effect to some certain extent. And tumor microenvironment with high neutrophils and low lymphocyte infiltration can promote blood tube formation, inhibit fine cell apoptosis, and promote tumor occurrence, resulting in poor prognosis. *Tanizaki et al.* substantiated that among individuals afflicted with locally advanced NSCLC subjected to Nivolumab treatment, PFS and OS experienced substantial elongation among those possessing elevated absolute neutrophil count and lymphocyte count before immunotherapy (63). Multiple investigations and meta-analyses have elucidated that a heightened neutrophil/lymphocyte ratio (NLR) correlates with an unfavorable prognosis in lung cancer patients (**Table 3**) (64–67, 87–89). Consequently, the association between NLR and immunotherapy response has been explored within the NSCLC patient cohort. In a study comprising 52 NSCLC patients, an elevated NLR exhibited an association with diminished OS (*P* < 0.001) and objective response rate (*P* = 0.013), albeit lacking correlation with PFS (*P* = 0.114). NLR demonstrated an AUC value of 0.738 for prognosticating the 10-month survival rate and 0.776 for predicting the efficacy of Nivolumab (66). Furthermore, the integration of NLR, among other variables, into a multivariate model substantially enhanced the prognostic capability of the model for predicting OS (68). *Bagley et al.* and *Fukui et al.* have corroborated the predictive worth of NLR in patients subjected to ICIs (69, 90). However, the optimal NLR threshold necessitates further establishment (70).

In addition to studies examining the potential utility of baseline NLR as a biological marker for immunotherapy response, various investigations have assessed the predictive and prognostic value of NLR during or post-treatment. *Suh et al.* made noteworthy observations regarding the median PFS and OS among patients with elevated NLR (≥ 5) six weeks after PD-1 blockade, demonstrating significantly shorter durations in comparison to patients with low NLR (median PFS: 1.3 vs. 6.1 months, *P* < 0.001; median OS: 2.1 vs. 14.0 months, *P* < 0.001) (71). Multivariate analysis confirmed that heightened NLR following treatment served as an independent prognostic indicator for OS (*P* = 0.003), indicating the potential of NLR six weeks after initiation of anti-PD-1 immunotherapy as a promising prognostic factor for patients with advanced NSCLC. Similarly, *Passiglia et al.* observed that a 20% or greater increase in NLR six weeks after Nivolumab treatment was associated with inferior OS (median OS: 8.7 vs. 14.6 months, *P* = 0.035) and TTP (5.2 vs. 10.3 months, *P* = 0.039) (61). The derived NLR (dNLR) may possess greater relevance than NLR, as it incorporates monocytes and other granulocyte subpopulations. Elevated dNLR has been linked to reduced survival in patients with multiple tumor types, including melanoma, pancreas, bladder, and renal cancer (72, 91–94). *Mezquita et al.* determined that a dNLR exceeding 3 represents the most suitable cutoff value for PFS and OS (73). Intriguingly, an inflammatory state (dNLR > 3) exhibited associations with shorter OS and durable clinical benefit among patients with advanced NSCLC receiving ICIs (95). Similarly, elevated dNLR (>3) demonstrated significant correlations with inferior PFS and OS (both *P* < 0.05) in patients with advanced NSCLC expressing at least 50% PD-L1 on tumor cells and treated with first-line Pembrolizumab (74). Although numerous studies have proposed the potential of peripheral blood cell count and its derivative indices as informative predictors of immunotherapy efficacy and prognosis, their clinical application remains limited due to considerations of peripheral blood cell count volatility and the absence of standardized research criteria.

#### 2.3.4 Serum neoplasm biological markers

Serum neoplasm biological markers assume a pivotal function in monitoring the efficacy of interventions in individuals afflicted with NSCLC, specifically those undergoing chemotherapeutic or targeted interventions. Several frequently evaluated serum neoplasm biological markers encompass carcinoembryonic antigen (CEA), neuron-specific enolase (NSE), cytokeratin fragment 21-1 (Cyfra21-1), and carbohydrate antigen (CA19-9) (96, 97). Under the simplicity associated with acquiring serum samples, extensive scrutiny has been devoted to these markers as prognosticators of the response to immunotherapy (**Table 3**). In a multicenter investigation encompassing 189 subjects with advanced NSCLC subjected to Nivolumab as a second- or later-line treatment, *Kataoka et al.* uncovered an association linking pre-immunotherapy CEA concentrations to PFS; a crucial threshold of 13.8 ng/ml for CEA was established (75). The cohort surpassing 13.8 ng/ml of CEA exhibited a significantly abbreviated PFS (*P* = 0.002). Conversely, no such correlation manifested between Cyfra21-1 serum levels and the aforementioned outcome. In contrast, *Shirasu et al.* identified Cyfra21-1 as a prognostic indicator for individuals with lung adenocarcinoma undergoing Nivolumab (76). Discrepancies in the outcomes can be ascribed to the limited sample sizes and variations in subgroup categorizations based on pathological classifications. Intriguingly, an additional study demonstrated that a minimal 20% reduction in CEA and Cyfra21-1 concentrations after immunotherapy correlated with enhanced durable clinical rate (DCR; *P* = 0.021 for CEA; *P* < 0.001 for Cyfra21-1), PFS (*P* = 0.028 for CEA; *P* < 0.001 for Cyfra21-1), and OS (*P* = 0.026 for CEA; *P* = 0.019 for Cyfra21-1) (98). Moreover, *Lang et al.* substantiated that individuals experiencing diminished levels of serum neoplasm markers (CEA, Cyfra21-1, CA19-9, and NSE) after immunotherapy demonstrated significantly protracted PFS and OS (77).

### 2.4 Gene mutations

Multiple investigations have documented the correlation between the expression of programmed death-ligand 1 (PD-L1) and driver mutations in NSCLC; nevertheless, the findings exhibit contradictoriness (10, 99–102). The recent meta-analysis hath manifested a considerable level of heterogeneity amidst the expression of PD-L1 and driver mutations (10, 103). In accordance with the NCCN guidelines, immunotherapy is recommended for patients with NSCLC who test negative for *EGFR* mutations and *ALK* rearrangements (3). Ergo, data about the clinical efficacy of immunotherapy in patients with NSCLC harboring driver mutations art constrained. Another meta-analysis hath disclosed that, in comparison to patients with *EGFR* mutations, those afflicted with *EGFR* wild-type NSCLC evince significantly protracted progression-free survival (*P* < 0.00001) and OS (*P* < 0.05) after immunotherapy with programmed cell death protein 1 (PD-1)/PD-L1 inhibitors (37). Through the employment of second-generation sequencing and exon sequencing methodologies, it hath also been ascertained that the median progression-free survival of patients with *EGFR* mutations is significantly briefer than that of patients sans *EGFR* mutations (51.0 versus 70.5 days, *P* = 0.0037) (104). *Cinausero et al.* hath assessed the impact of *KRAS* mutations on the effectiveness of anti-PD-1 immunotherapy (105). The neoplastic deoxyribonucleic acid (DNA) of 47 patients was sequenced, and 43% of these individuals did present with *KRAS* mutations. An observable disparity in the frequency of *KRAS* mutations hath been observed betwixt responders and non-responders. The PFS and OS of patients with *KRAS* mutations hath been superior to that of patients with wild-type *KRAS* (*P* = 0.032, 0.010 separately). Notwithstanding, a comprehensive retrospective study hath debunked any association between *KRAS* mutations and the effectiveness of immunotherapy in a cohort of 328 patients with advanced NSCLC (106).

The effectiveness of immunotherapy in NSCLC has been examined concerning *TP53* mutations. Non-synonymous *TP53* mutations were observed in 57% (41 out of 65) of NSCLC individuals. Following a median follow-up period of 15.2 mos, individuals with *TP53* mutations demonstrated a median OS of 18.1 months (95% CI, 6.6 – not reached), as opposed to 8.1 months for individuals with wild-type *TP53* (95% CI: 2.2 – 14.5; *P* = 0.04). Patients with *TP53* mutations also exhibited significantly longer median PFS (4.5 vs. 1.4 months, *P* = 0.03) and a higher ORR (51.2% vs. 20.7%; *P* = 0.01) in comparison to patients without *TP53* mutations. Multivariate analysis disclosed an independent correlation between *TP53* mutations and extended OS (*P* = 0.009) (107). Interestingly, *Shi et al.* have also found that patients with *TP53/KMT2C* co-mutation would get longer PFS and greater DCB (*P* = 0.033) when undergoing ICIs (108). A study involving 78 Chinese NSCLC individuals revealed that those with *FAT1* mutations exhibited higher rates of clinical benefit and objective response rates in comparison to individuals without *FAT1* mutations (71.4% vs. 22.7%, *P* = 0.01; 57.1% vs. 15.2%, *P* = 0.02) after undergoing ICIs. Furthermore, the loss of copy numbers in specific chromosome 3p fragments containing the tumor suppressor gene *TIGA9* and several chemokine receptor genes were strongly associated with unfavorable clinical outcomes (6-month survival rate: 0% vs. 31%; *P* = 0.012) (104). Elevated PD-L1 expression has been linked to deficiencies in DNA repair through homologous recombination (109). Consequently, a study explored the methylation status of the *Rad51B* promoter (*RAD51B^me^*), a crucial mediator of homologous recombination, PD-L1 expression, and the effectiveness of immunotherapy. *RAD51B^me^* levels were markedly higher in PD-L1-expressing individuals in comparison to PD-L1-negative individuals (*P* < 0.05). Furthermore, *RAD51B^me^* levels were significantly associated with the response to ICIs, suggesting its potential as a predictive indicator for the efficacy of immunotherapy in NSCLC (110). Although ICIs have demonstrated efficacy in NSCLC, the benefits of ICIs for individuals with positive driver gene mutations remain inconclusive. Targeted therapy is expected to continue playing a role in the treatment of NSCLC individuals with positive driver gene mutations in the future.

## 3 Combined biological markers

### 3.1 Radiology and radiomics

Radiomics encompasses the systematic assessment of tumor phenotype through the utilization of radiological images and bioinformatics tools. Its primary objective is to construct clinical models that enable the evaluation of tumor heterogeneity and the microenvironment in a high-throughput manner. Notably, multiple investigations have provided evidence that the radiological attributes of the peritumoral region hold potential as indicators of survival outcomes in individuals afflicted with lung cancer (111–113).

Artificial intelligence (AI)-assisted enhanced computed tomography (CT) was employed for the analysis of 1055 primary and metastatic lesions derived from patients diagnosed with advanced melanoma and NSCLC who underwent anti-PD-1 therapy. The objective of this study was to ascertain non-invasive biomarkers that could predict the response to treatment (114). In the case of NSCLC, the field of imageology revealed a highly significant marker (the highest AUC value was 0.83, *P* < 0.001), and a similar trend was observed in melanoma lymph nodes (AUC value = 0.64; *P* = 0.001). The pooled analysis yielded a maximum AUC value of 0.76 (*P* < 0.001) for the prediction of immunotherapy response, and it also highlighted a 24% disparity in the 1-year survival rate (*P* = 0.02). These findings suggest that radiological characteristics observed in CT imaging have the potential to serve as non-invasive biomarkers for the prediction of immunotherapy response. In a separate investigation conducted by Khorrami et al., machine learning techniques were employed to examine alterations in radiation texture parameters obtained from internal and external CT scans of tumor lesions, both before and after 2-3 cycles of ICIs therapy (DelRADx) (115). The retrospective analysis encompassed 139 NSCLC patients recruited from two research centers, with a division into a discovery group (D1 = 50) and two independent verification cohorts (D2 = 62 and D3 = 27). Through the implementation of machine learning methodologies, texture parameters within and surrounding the tumor were extracted, facilitating the computation of relative differences between pre-ICIs and post-ICIs treatment conditions. DelRADx proved capable of predicting both ICI response and overall survival (OS) among NSCLC patients. The linear discriminant analysis (LDA) classifier achieved AUC values of 0.88 ± 0.08 (D1), 0.85 (D2), and 0.81 (D3), effectively discriminating responders from non-responders. Moreover, the delta-radiomic risk score (DRS) demonstrated a significant correlation with OS (*P* = 0.0011; C-index = 0.72), thereby suggesting the potential utility of DelRADx as a tool for identifying NSCLC patients likely to exhibit a positive response to immunotherapy.

An analysis was undertaken encompassing 32 patients who had been diagnosed with NSCLC before undergoing treatment with Nivolumab. The utilization of 18F-FDG PET/CT scans facilitated this examination. The findings reveal a substantial disparity in the maximum standardized uptake value (SUVmax) between individuals who responded positively (N = 21) and those who did not (N = 11) (48.97 vs. 20.85, *P* = 0.002) (116). Furthermore, non-responders exhibited a tendency towards elevated tumor metabolic volume (MTVwb) and total lesion glycolysis (TLGwb) in comparison to responders. Likewise, a study involving one hundred and ninety-four patients with histologically confirmed stage IIIB-IV NSCLC investigated the potential of 18F-FDG PET/CT images acquired before treatment to identify a radiomics signature capable of predicting the response to immunotherapy (117). The resulting multiparametric radiomics signature (mpRS) demonstrated promising prognostic capabilities for durable clinical benefit (DCB), with respective AUC values of 0.86, 0.83, and 0.81 in the training, retrospective, and prospective test cohorts. Moreover, the nomogram models attained C-indexes of 0.74, 0.74, and 0.77 for prognosticating PFS, along with C-indexes of 0.83, 0.83, and 0.80 for predicting OS across the three cohorts. Furthermore, *Seban et al.* discovered through the analysis of FDG PET/CT that a total metabolic tumor volume (TMTV) surpassing 75 cm^3^ was linked to diminished overall survival and the absence of DCB in patients with advanced NSCLC who received treatment with ICIs (95). The study also established a significant correlation between high TMTV and unfavorable PFS and OS (both *P* < 0.05) in patients with advanced NSCLC who underwent first-line Pembrolizumab treatment (74). These findings suggest the potential usefulness of 18F-FDG PET/CT imaging parameters as predictive indicators for the efficacy of immunotherapy in patients with NSCLC. However, the clinical implementation of these parameters has been impeded by the redundancy and lack of replicability of several image features (118). Several ongoing prospective studies (NCT03304639, NCT03387761, and NCT03237780) are presently assessing the value of radiology and radiomics in predicting the response to ICIs.

### 3.2 Prediction models

#### 3.2.1 Metabolic score

A composite prognostic biological marker employing dNLR and TMTV exhibits promising prospects in the domain of NSCLC. The metabolic scoring system classifies patients into three distinct categories: favorable prognosis (TMTV ≤ 75 cm3, dNLR ≤ 3), intermediate prognosis (TMTV > 75 cm^3^ or dNLR > 3), and unfavorable prognosis (TMTV > 75 cm^3^ and dNLR > 3). In a retrospective analysis encompassing 109 patients with advanced NSCLC who underwent ICIs, the metabolic score showcased noteworthy variances in terms of median OS and median PFS across the aforementioned categories (*P* < 0.001) (92). The favorable prognosis group exhibited the lengthiest median OS of 35.0 months, followed by the intermediate prognosis group (12.5 months), and finally, the unfavorable prognosis group (2.4 months). Median PFS values were recorded as 9.8, 2.7, and 1.4 months, respectively (*P* < 0.001). Notably, the metabolic score exhibited a correlation with ICI response, particularly in terms of durable clinical (*P* = 0.003) (**Table 4**). Within a multicenter study comprising 63 NSCLC patients possessing a PD-L1 tumor proportion score (TPS) ≥ 50, who were administered first-line Pembrolizumab and fluorodeoxyglucose positron emission tomography/computed tomography (FDG PET/CT), the favorable prognosis group exhibited a median OS of 17.9 months, in contrast to 13.8 and 6.6 months for the intermediate and unfavorable prognosis groups, respectively (74). Median PFS values were reported as 15.1, 5.2, and 1.9 months. The unfavorable prognosis group demonstrated associations with the DCR and ORR (*P* < 0.05). These findings lend support to the potential utilization of the metabolic score as a prognostic factor for NSCLC patients undergoing ICI treatment. Nonetheless, it is imperative to conduct prospective studies to validate the prognostic worthiness of this scoring system, given the retrospective nature of the existing evidence.

**Table 4:** Clinical outcomes of NSCLC patients received anti-PD-1/anti-PD-L1 agents according to different prediction models.

Model	Patients (n)	Groups	(median)PFS (month)	(median)OS (month)	Ref
LIPI	126 (Test)	Good **vs.** Intermediate **vs.** Poor	6 vs. 3 vs. 1	34 vs. 10 vs. 3	(73)
	305 (Validation)	Good **vs.** Intermediate **vs.** Poor	6.7 vs. 4.2 vs. 3.6	14.2 vs. 10.0 vs. 6.2	
	431 (Pool)	Good **vs.** Intermediate **vs.** Poor	6.3 vs. 3.7 vs. 2.0	16.5 vs. 10.0 vs. 4.8	
Metabolic Score	80	Good **vs.** Intermediate **vs.** Poor	9.8 vs. 2.7 vs. 1.4	35 vs. 12.5 vs. 2.4	(95)
Metabolic Score	63	Good **vs.** Intermediate **vs.** Poor	15.1 vs. 5.2 vs. 1.9	17.9 vs. 13.8 vs. 6.6	(74)
iSEND Model	159	Good **vs.** Intermediate **vs.** Poor	17.4 vs. 5.3 vs. 2.8	NR vs. 23.4 vs. 7.1	(119)
iSEND Model	439	Good **vs.** Intermediate **vs.** Poor	6.5 vs. 4.0 vs. 1.9	23 vs. 13.4 vs. 4.5	(120)
EPSILoN Score	154	Good **vs.** Moderate **vs.** Poor	10.2 vs. 4.9 vs. 1.7	NA	(121)
EPSILoN Score	193	Good **vs.** Intermediate **vs.** Poor	6.0 vs. 3.8 vs. 1.9	24.5 vs. 8.9 vs. 3.4	(122)
LIPI	216	Good **vs.** Intermediate **vs.** Poor	6.1 vs. 2.3 vs. 2.1	24.2 vs. 14.5 vs. 9.3	(123)
Dynamic LIPI	179	Good vs. Intermediate vs. poor	8.4 vs. 2.1 vs. 1.4	NA	
LIPI	153	Good **vs.** Intermediate **vs.** Poor	6.6 vs. 5.1 vs. 2.8	20.8 vs. 7.3 vs. 3.4	(124)
LIPI	1489	Good **vs.** Intermediate **vs.** Poor	4.2 vs. 2.7 vs. 1.4	18.4 vs. 11.3 vs. 4.5	(125)
LIPI	1368	Good **vs.** Intermediate **vs.** Poor	5.7 vs. 3.5 vs. 2.1	15.6 vs. 8.9 vs. 4.5	(126)

NA, not available; NR, not reached; iSEND Model, the NLR and Delta NLR model; EPSILoN Score, ECOG PS, smoking, liver metastasis, LDH, and NLR score; LIPI, lung immune prognostic index.

#### 3.2.2 iSEND model

The NLR and Delta NLR (iSEND) model was initially introduced within the context of predicting the clinical efficacy of Nivolumab among patients diagnosed with NSCLC (119). An analysis conducted retrospectively on a cohort of 139 patients with locally advanced NSCLC, who had received second-line Nivolumab treatment, yielded correlations between sex, ECOG score, NLR, as well as pre-treatment and post-treatment changes in NLR, and PFS. The aforementioned variables were subsequently integrated into the iSEND model. By the iSEND model, patients were stratified into low-, medium-, and high-risk groups. The median follow-up period extended to 11.5 months. Within the low-risk group, the rates of 3, 6, 9, and 12-month PFS were observed at 78.4%, 63.7%, 55.3%, and 52.2%, respectively. Correspondingly, the medium-risk group experienced rates of 79.4%, 44.3%, 25.9%, and 19.2%, while the high-risk group exhibited rates of 65%, 25.9%, 22.8%, and 17.8%. As for the iSEND model’s ability to predict 3, 6, 9, and 12-month PFS, the respective AUC values amounted to 0.718, 0.74, 0.746, and 0.774. Significantly, disease progression within the high-risk group at 12 ± 2 weeks exhibited a strong correlation (*P* < 0.0001). These findings convey that the iSEND model can serve as a valuable tool in forecasting the prognosis of locally advanced NSCLC patients post Nivolumab treatment. In a subsequent investigation featuring a median follow-up duration of 18.2 months, patients classified as low-risk demonstrated notably superior OS rates in comparison to those deemed high-risk (*P* < 0.0001) (120). Furthermore, the prognostic capability of the iSEND model was assessed relative to PD-L1 expression levels. The time-dependent mortality rates in the iSEND low-risk group (N = 119) and the PD-L1 TPS = 0% group (N = 47) were found to be 0.75 vs. 0.53 at 12 months (*P* = 0.01) and 0.85 vs. 0.46 at 18 months (*P* = 0.03), respectively. Nevertheless, no significant distinction was observed in terms of the prognostic value between the iSEND model and PD-L1 TPS ≥ 50%. As such, the iSEND model holds potential as a prognostic factor applicable to patients with locally advanced NSCLC following Nivolumab therapy (**Table 4**).

#### 3.2.3 EPSILoN score

The EPSILoN scoring system, which encompasses the ECOG PS, smoking history, liver metastasis, lactate dehydrogenase (LDH), and NLR, has been proposed as a prognostic tool for predicting the response to immunotherapy in individuals diagnosed with NSCLC (**Table 4**). Within a cohort comprising 154 patients with locally advanced NSCLC, who were administered second-line or later-line anti-PD-1 therapy, the ECOG score, smoking history, liver metastasis, LDH, and NLR demonstrated significant associations with both PFS and OS. Consequently, these aforementioned factors were incorporated into the EPSILoN scoring system. The patients were subsequently stratified into categories denoting favorable, moderate, and unfavorable prognoses based on their EPSILoN scores. The resulting median PFS values for each category were observed to be 10.2, 4.9, and 1.7 months, respectively (*P* < 0.001) (121). During the validation study of the EPSILoN scoring system among patients with locally advanced NSCLC who underwent anti-PD-1 immunotherapy, participants were divided into high, moderate, and low-risk groups (122). The corresponding PFS durations were 6.0, 3.8, and 1.9 months, respectively (*P* < 0.001), while the OS values were 24.5, 8.9, and 3.4 months (*P* < 0.001). These results affirm the prognostic significance of the EPSILoN scoring system for individuals with NSCLC who are receiving ICIs.

#### 3.2.4 LIPI score

The lung immune prognostic index (LIPI) has been postulated as a novel categorical hematological biological marker to select individuals diagnosed with NSCLC who are suitable candidates for PD-1/PD-L1 therapy. LIPI integrates the dNLR and LDH to categorize patients into three distinct prognostic subsets. These subsets are demarcated by the subsequent thresholds: dNLR ≤ 3 and LDH ≤ upper limit of normal (ULN) to identify the low-risk cohort, dNLR ≥ 3 or LDH ≥ ULN to classify the moderate-risk group, and dNLR ≥ 3 and LDH ≥ ULN to allocate patients to the high-risk category (**Table 4**) (123–126). The work carried out by *Mezquita et al.* entailed a seminal investigation that established a noteworthy association between LIPI and treatment outcomes in patients with advanced NSCLC undergoing ICIs, namely Nivolumab, Pembrolizumab, Atezolizumab, Durvalumab, or Durvalumab plus Ipilimumab (73). The study demonstrated that dNLR values exceeding 3 and LDH levels surpassing the ULN were independently linked to OS. The high-risk, intermediate-risk, and low-risk groups exhibited median OS durations of 3, 10, and 34 months, respectively (*P* < 0.001). Median PFS values were measured at 2.0, 3.7, and 6.3 mos (*P* = 0.001), respectively. Moreover, the disease control rate displayed a positive correlation with dNLR values surpassing 3 and LDH levels exceeding the ULN (*P* = 0.004). Nevertheless, the prognostic value of the LIPI score failed to attain statistical significance within the chemotherapy cohort. The investigators concluded that pretreatment LIPI, encompassing dNLR values greater than 3 and LDH levels surpassing the ULN, was indicative of unfavorable outcomes in patients receiving ICIs, thereby suggesting its potential utility in identifying individuals unlikely to derive benefit from ICIs.

In a subsequent investigation conducted across multiple centers, the prognostic and predictive value of the LIPI score was examined among patients diagnosed with advanced NSCLC who were undergoing Nivolumab monotherapy (124). An insufficient LIPI score demonstrated a significant correlation with unfavorable OS according to both univariate analysis (*P* < 0.0001) and multivariate analysis (*P* < 0.0001). Although a noticeable association with diminished PFS was observed in the univariate analysis (*P* = 0.03), this correlation failed to reach statistical significance in the multivariate analysis (*P* = 0.09). Moreover, a low LIPI score displayed a statistically significant relationship with a reduced DCR based on both univariate analysis (*P* = 0.001) and multivariate analysis (*P* = 0.005). *Sorich et al.* conducted an extensive aggregated examination, assimilating data from the BIRCH, FIR, OAK, and POPLAR clinical trials, encompassing a total of 1489 patients who received Atezolizumab. The analysis unveiled a noteworthy correlation between the Lymphocyte Monocyte Ratio (LMR) score and the OS, PFS, and response rates, all possessing a level of significance below *P* < 0.001. Within the low-risk, intermediate-risk, and high-risk cohorts, the durations of median OS were found to be 18.4, 11.3, and 4.5 months, respectively (125). Importantly, the LMR score also exhibited correlations with survival (*P* < 0.001) and response rates (*P* = 0.005) in patients subjected to docetaxel treatment. In an additional pooled analysis comprising 11 clinical trials involving patients with metastatic NSCLC, a high LMR score displayed a favorable association with overall survival. Specifically, patients with a high LMR score demonstrated an estimated median survival of 15.6 months, whereas those with a low score had a median survival of 4.5 months (*P* < 0.001) (12). Analogous associations between elevated LMR scores and enhanced survival outcomes were observed among patients who underwent chemotherapy. In this context, patients with a high LMR score experienced a protracted period of survival in comparison to those with a low score, with an estimated median survival of 10.4 mos versus 5.3 mos (*P* < 0.001). Thus, the pretreatment LMR score exhibits promise as a valuable instrument for identifying patients who are likely to derive benefits from ICIs and chemotherapy.

## 4 Conclusion and future perspectives

The clinical application of PD-1/PD-L1 inhibitors in patients diagnosed with NSCLC has achieved unprecedented success in terms of enhancing long-term outcomes. However, the relatively low response rates, elevated treatment costs, and notable likelihood of immune-related adverse reactions necessitate an urgent quest for effective predictive and prognostic biological markers. At present, PD-L1 expression and TMB appear to hold the most promising potential as biological markers for predicting the response to immunotherapy. Nonetheless, these single biological markers require comprehensive exploration and optimization in various aspects due to certain limitations identified in specific cases (**Table 5**). Furthermore, emerging and promising biological markers encompass hematological biological markers, driver mutations, radiology, and radiomics. Going forward, it is imperative to standardize the diverse range of biological markers, leverage omics technologies to expedite the identification of robust biological markers, examine the feasibility of employing combination biological markers, and harness the capabilities of computer algorithms and AI technologies to establish innovative prognostic models. In the context of this review, it appears that prediction models incorporating multiple factors hold greater promise as tools for prognosticating the effectiveness of immunotherapy in NSCLC. During clinical trials, stratified analyses can be conducted based on the factors included in these models to identify subgroups that are more likely to benefit from immunotherapy.

**Table 5:** Some advantages and disadvantages of single biological markers for predicting immunotherapy in NSCLC.

Characteristics	Advantages	Disadvantages
PD-L1 expression	● NCCN guidelines recommend testing, commonly use in clinic due to strong forecasting efficiency. ● The detection in convenient and moderate cost.	● Part of the tumor is difficult to reflect the whole. ● Easily to be affected by sampling time and location, and is not completely related to the curative effect of treatment.
TMB	● Commonly used in clinic and easy to detect. ● The performance of prediction is better.	● The amount of samples needed for detection is larger. ● Long testing period, high cost and lack of unified testing standards.
CD8+ T Cells	● It has a broad prospect and may become a method of tumor treatment.	● Fewer clinical applications and lack of large-scale clinical trials.
ctDNA	● The change is prior to the imaging change, which is helpful to judge the immune effect in advance. ● Sampling is simple.	● Not all NSCLC patients can be detected. ● The cost of testing is higher.
Blood cell count	● Can be used as a dynamic monitoring index due to the sampling is convenient and the testing cost is low.	● Easily disturbed by other factors such as inflammation of the body.
Gene mutation	● The detection is convenient and the cost is moderate.	● Lack of large clinical trials on the relationship between rare driving gene mutations and immune efficacy.

## Author contributions

DH and CL conceived of the review and edited the manuscript. SL, HW, and HT analyzed the data and drafted the manuscript. ZX, and MW contributed to the data collection. All authors read and approved the final manuscript. All authors contributed to the article and approved the submitted version.
